# Evaluation of strategies to modify Anti-SARS-CoV-2 monoclonal antibodies for optimal functionality as therapeutics

**DOI:** 10.1371/journal.pone.0267796

**Published:** 2022-06-03

**Authors:** Robert V. House, Thomas A. Broge, Todd J. Suscovich, Doris M. Snow, Milan T. Tomic, Genevieve Nonet, Kamaljit Bajwa, Guangyu Zhu, Zachary Martinez, Kyal Hackett, Christopher G. Earnhart, Nicole M. Dorsey, Svetlana A. Hopkins, Dalia S. Natour, Heather D. Davis, Michael S. Anderson, Melicia R. Gainey, Ronald R. Cobb

**Affiliations:** 1 Ology Bioservices, Frederick, MD, United States of America; 2 SeromYx Systems, Cambridge, MA, United States of America; 3 Research and Development, Ology Bioservices, Inc., Alameda, CA, United States of America; 4 US Department of Defense, Joint Program Executive Office for Chemical, Biological, Radiological, Nuclear Defense (JPEO-CBRND), Washington, DC, United States of America; 5 Logistics Management Institute, Tysons, VA, United States of America; 6 Battelle Biomedical Research Center, West Jefferson, Columbus, Ohio, United States of America; 7 Process Development, Ology Bioservices, Alachua, FL, United States of America; University of Hail, SAUDI ARABIA

## Abstract

The current global COVID-19 pandemic caused by severe acute respiratory syndrome coronavirus 2 (SARS-CoV-2) has resulted in a public health crisis with more than 168 million cases reported globally and more than 4.5 million deaths at the time of writing. In addition to the direct impact of the disease, the economic impact has been significant as public health measures to contain or reduce the spread have led to country wide lockdowns resulting in near closure of many sectors of the economy. Antibodies are a principal determinant of the humoral immune response to COVID-19 infections and may have the potential to reduce disease and spread of the virus. The development of monoclonal antibodies (mAbs) represents a therapeutic option that can be produced at large quantity and high quality. In the present study, a mAb combination mixture therapy was investigated for its capability to specifically neutralize SARS-CoV-2. We demonstrate that each of the antibodies bind the spike protein and neutralize the virus, preventing it from infecting cells in an *in vitro* cell-based assay, including multiple viral variants that are currently circulating in the human population. In addition, we investigated the effects of two different mutations in the Fc portion (YTE and LALA) of the antibody on Fc effector function and the ability to alleviate potential antibody-dependent enhancement of disease. These data demonstrate the potential of a combination of two mAbs that target two different epitopes on the SARS-CoV2 spike protein to provide protection against SARS-CoV-2 infection in humans while extending serum half-life and preventing antibody-dependent enhancement of disease.

## Introduction

In the past decades, two known pathogenic human coronaviruses, severe acute respiratory syndrome (SARS-CoV) and Middle East respiratory syndrome CoV (MERS-CoV), have been reported to damage the respiratory tract and cause high morbidity and mortality [1]. Severe acute respiratory syndrome coronavirus 2 (SARS-CoV-2), the causative agent of COVID-19, is a newly discovered coronavirus that was first reported in the city of Wuhan, Hubei province, China in December 2019 [2]. The resulting pandemic has made the development of therapeutics, vaccines and diagnostics an urgent global priority [3–7]. Initial work identified that this virus uses the angiotensin-converting enzyme 2 (ACE2) from bats, civet cats, swine, non-human primates and humans as a receptor [3,8,9]. The initial reports from China and elsewhere note that although most COVID-19 cases present mild to moderate pathology, approximately 20% of the cases are severe [10,11].

Just as the development and use of mAbs have revolutionized the treatment of cancer, they are increasingly recognized as potential therapeutic treatments for infectious diseases. However, the lack of detailed knowledge of the correlates of protection has hindered the development of effective mAb therapeutics for the treatment of infectious disease. Of the almost 80 approved mAb therapeutics, only four have been approved for the treatment of infectious diseases. These include raxibacumab and obliltoxaximab for the treatment of inhalation anthrax [12], palivizumab for the prevention of respiratory syncytial virus in high risk infants [13], and ibalizumab for treatment of HIV infection [14]. For many infectious disease targets, neutralization is often the primary means by which antibody candidates are selected for clinical development, even if neutralization has not been linked to efficacy. However, similar to mounting importance of extra-neutralizing antibody functions in vaccine-mediated protection from infection, extra-neutralizing antibody functions are increasingly recognized as critical in mAb-mediated protection from infection [15–19], even for neutralizing antibodies [20–22].

In contrast to their protective role, antibodies have been shown to exacerbate disease through a phenomenon known as antibody-dependent enhancement (ADE) of disease or infection. It has been shown that antibodies can facilitate the entry of viruses into target cells even when the cell lacks the expression of the normal viral receptor. Antibodies have been shown to facilitate entry of both SARS virus and MERS virus into FcγR2-expressing cells [23]. In addition, ADE of acute lung injury has been documented in animal models of SARS and MERS virus infection [24,25]. Engagement of the FcγRs is required for antibody effector functions, such as antibody-dependent cellular cytotoxicity (ADCC) and antibody-dependent cellular phagocytosis (ADCP) [26]. The Fc portion of antibodies have been optimized using multiple approaches in attempts to increase binding affinity to selected FcγR [27].

While the precise role of additional neutralizing antibody functions in SARS-CoV-2 infection has not yet been elucidated, previous studies have demonstrated the importance of these functions. In a mouse model of SARS virus infection, passive immunization with SARS-CoV-2 antiserum demonstrated greater efficacy than immunization with rabbit anti-SARS-CoV-2 antiserum. It was determined that the increased efficacy was mediated by phagocytic cells (primarily monocyte-derived infiltrating macrophages and alveolar macrophages), suggesting that ADCP may be critical in driving protection [28]. Recruitment of monocytes to the lung has been shown to be necessary for protection from lethal infection of SARS virus in other mouse models [29]. Similarly, neutrophils are needed for an effective immune response against pulmonary rat coronavirus infection but also contribute to the underlying lung pathology, suggesting a complicated role for neutrophils in coronavirus infection [30]. Following mouse hepatitis virus 1 infection of mice, the natural killer (NK) cell response helps to minimize the severity of the disease [31]. Human antibodies to naturally acquired coronavirus 229E have ADCC activity, suggesting that NK cell-mediated function may also be important for resolution of coronavirus infection [32]. In contrast, complement activation has been linked to lung pathology following SARS virus infection and in mouse models of MERS virus, suggesting that excessive antibody-dependent complement activation could be detrimental and may be underlying the cytokine storm driving severe lung damage [33–36].

While Fc optimization has focused heavily on the gain-of-function modifications, in certain situations it can be beneficial to eliminate antibody Fc function. Under these conditions, Fc engagement of the receptors on effector cells or engagement of C1q is not desired, because it can lead to undesired killing of the biologically-important cells expressing the receptor or recruitment of drug-conjugated antibodies to off-target cells [37,38]. A double mutation in the Fc region of an IgG1 antibody, Leu234Ala and Leu235Ala (LALA) has been shown to eliminate detectable binding to FcγRIIa, Fcγ RI, IIB, and IIC for IgG1 and IgG4 [39–41]. The LALA mutations have been tested in human clinical trials with minimal adverse effects [42]. Currently, LY-CoV016 that contains the LALA mutation is currently in clinical development in combination with another anti-SARS-CoV-2 antibody (LY-CoV555) and has shown clinical success. Recently, Winkler et al [43] presented data implying that an intact Fc effector function was required for optimal therapeutic protection against SARS-CoV-2. Data from this manuscript demonstrated that clinical protection with MAB2381 was only partially Fc-dependent when administered post-exposure yet virological protection was not lost with the LALA-PG mutation. The authors concluded that the differences observed in the therapeutic activity of the different antibodies would be due to differential pharmacokinetics, bioactivity or bioavailability.

In addition to modifying antibody effector functions optimizing efforts have also included improvements in maintaining antibody circulation *in vivo*. Degradation of immunoglobulins is regulated by its interaction with the neonatal Fc receptor (FcRn). At physiologic pH of the extracellular environment IgG has weak affinity for the FcRn which results in its release from the FcRn back into circulation [44]. The pH-dependent binding is regulated by protonation of His310, 435, 436 in the Fc at low pH which enables the antibody to bind to Glu117, Glu132 and Asp137 in the FcRn [45,46]. Phage display libraries were used to identify potential locations where changes in the amino acid sequence would result in increased antibody half-life. These studies identified Met252Tyr, Ser254Thr and Thr256Glu mutations, termed YTE, and resulting in a 10-fold slower dissociation rate of Fc and FcRn [47]. Overall, the YTE mutations increased the serum half-life 4–5 fold as compared to wild type without having deleterious effects to antigen binding [48,49].

In the present study, we evaluated two different anti-SARS-CoV-2 antibodies that have been previously shown to bind to the spike protein and block the Receptor Binding Domain (RBD) binding to the ACE2 receptor [50]. Different Fc variants that contained the wild type Fc sequences, the YTE mutations alone or the YTE plus LALA mutations were evaluated for their ability to bind the RBD of the spike protein and the effects of the mutations on neutralization of the virus to infect cells. Additionally, the study provides data based on the cell-based evaluation of these mutations on the possible effector functions of these antibodies. Two of these antibodies (2130 YTE-LALA and 2381 YTE-LALA) which have been shown to bind to non-overlapping epitopes on the spike protein [50] and a combination of these two antibodies (ADM03820) were evaluated for their ability to neutralize a panel of SARS-CoV-2 spike protein variants and wild type virus that included the United Kingdom, South African and Brazilian variants. Taken together, the data demonstrate that the YTE and YTE-LALA mutations did not have any negative effect on the binding of the antibodies to the RBD and were equally efficacious in neutralizing the virus and preventing it from infecting cells and no antibody-dependent enhancement of infection was observed with any of the different mAbs.

## Materials and methods

### Monoclonal antibodies

The mAb sequences and numbering system were obtained from Dr. James Crowe’s laboratory and have been described previously [50,51]. A list of the antibodies that we evaluated are presented in Table 1. The antibody sequences and YTE and YTE-LALA mutations were cloned into antibody expression vectors and subsequently used to stably transfect CHO GS null cells. The mAb were grown at 37° C using BalanCD media. mAb were purified in a similar fashion as described previously [52].

**Table 1:** List of monoclonal antibodies evaluated.

Sample
mAb 2130 Wildtype
mAb 2130 YTE
mAb 2130 YTE LALA
mAb 2819 Wildtype
mAb 2819 YTE
mAb 2819 YTE LALA

### ELISA for spike protein binding

Binding to the SARS-CoV-2 spike protein were evaluated using His-tagged materials (S1, RBD, and S2) purchased from ACRObiosystems (San Jose, CA). These materials were used to coat Nunc 96-well Immulon 4HBX plates (100 μL) at a concentration of 1 μg/mL. The plates were incubated 12–24 hours at 2–4° C. The plates were then washed three times with Wash Buffer (WB; 1XPBS containing 0.05% Tween-20). The wells were then blocked using 200 μL of Blocking Buffer (BB; 1XPBS containing 3% BSA). The plates were incubated 1–4 hours at room temperature. The wells were then washed three times using WB. Positive control antibodies or test samples were diluted in Assay Dilution Buffer (ADB; 1XPBS, 0.05% Tween-20 and 1% BSA) and 100 μL was added to each well. The plates were incubated for 60±10 minutes at room temperature on a shaking platform. The plates were then washed three times using WB. The secondary goat anti-human IgG(Fc)-HRP (Abcam, Cambridge, MA) was diluted 1:25,000 in ADB and 100 μL was added to each well. The plates were incubated for 60±10 minutes at room temperature on a shaking platform. Finally, 100 μL of Stop Solution (0.2M H2SO4) was added to the plates and the plates were read at 450–630 nm using a Molecular Devices SpectraMax 384 Plus (Promega, Madison, WI). Each sample was tested in triplicate.

### Microneutralization potency

The SARS-CoV-2 microneutralization potency assay quantifies the neutralizing capacity of a human mAb. For the assay, serial dilutions of the mAb were pre-incubated with the SARS-CoV-2 for 1 hour at 37° C. The mAb/virus mixtures were then added to a 90 to 100% confluent monolayers of VERO E6 cells in 96-well microplates and incubated for two days at 37° C and 5% CO_2_. Following incubation, the inoculum was removed and monolayers were incubated for at least 30 minutes in cold 80% acetone to allow cell fixation.

For the *in situ* ELISA (Battelle Memorial Institute, Patent Application Number 63/014,551), the fixed VERO E6 monolayer was incubated with commercial anti-coronavirus protein antibodies for 1 hour at 37° C. The plate was washed and then a commercial horseradish peroxidase secondary antibody was added and the plate incubated for 1 hour at 37° C. The plate was washed and a commercial ABTS substrate and stop solution were used per manufacturer’s instructions and the optical density at 405 nm with a 490 nm reference filter was obtained. Each plate contained a virus-only control and a cell culture control. The 50% inhibitory concentration (IC_50_) and 80% inhibitory concentration (IC_80_) for each mAb was determined by logistic model. Control antibodies were procured from commercial sources and were tested for neutralization in the potency assay.

### Virus assays

Numerous single point mutations and combinations of mutations of the spike protein displayed on pseudovirus as well as wild type variants of concern (VOCs) were evaluated by the Division of Microbiology and Infectious Diseases, part of the National Institute of Allergy and Infectious Disease. The panel includes variants with different substitutions such as E484K, F490S, Q493R and S494P. Briefly, the pseudovirus containing each of the variants were generated by transfecting HEK293T cells with CoV-2 S, luciferase, and gag-pol plasmids and harvest the S-pseudovirus after 48 hours. The pseudovirus were evaluated for infectivity post infection. Positive control antibodies against wild type (614G) were used to determine the appropriate range for the neutralization assays. For the neutralization assays, the pseudoviruses were combined with each of the antibodies or the combination of antibodies and then used to inoculate HEK293T ACE2/TMPRSS2 cells. Luciferase levels were measured 48 hours post infection. Each variant and antibody were evaluated in at least two independent experiments with independent operators.

### Fc effector function assays

Each mAb was tested in an eight-point dilution curve for each assay, with each antibody tested in two independent experiments. For assays in which primary cells were used as effector cells, cells were isolated from at least two different human donors. For assays utilizing cell lines as effector cells, each assay was repeated in two independent experiments.

### Antigen-specific FcγR binding

The Fc receptor Luminex array uses fluorescently coded microspheres to capture up to 500 antigen specificities simultaneously and profile the effector capacity of each antigen specificity by determining the ability of these antigen-specific antibodies to interact with Fc receptors. Stabilized SARS-CoV-2 spike trimer (LakePharma) was covalently coupled to Magplex Luminex beads via a two-step carbodiimide reaction. The beads were activated for 30 minutes at room temperature using 100 mM monobasic sodium phosphate, pH 6.2 with 5 mg/mL Sulfo-NHS (N-hydroxysulfosuccinimide, Pierce, A39269) and 5 mg/mL ethyl dimethylaminopropyl carbodiimide hydrochloride (EDC), then washed with 50 mM MES, pH 5.0, and incubated with 25 μg of antigen in 50 mM MES, pH 5.0 for two hours on a rotator. The coupled beads were blocked 5% BSA-PBS. After blocking, coupled beads were washed in PBS-Tween, resuspended in PBS, and stored at 4˚C. Coupled beads were diluted to a concentration of 100 microspheres/μL and incubated with diluted antibody (diluted in PBS), and incubated at room temperature for 2 hours, shaking at 800 rpm. Following a wash with 0.1% BSA in PBS-Tween, the bound antibodies were subsequently stained with PE-labeled tetramerized recombinant Fc receptors (FCGR2A 131H/R, FCGR2B, FCGR3A 158V/F, FCGR3B, and FcRN) for 1 hour at room temperature, shaking at 800 rpm. Fluorescence was analyzed using a Stratedigm S1000EON. The data are reported as the median fluorescence intensity of PE for a specific bead channel. The mAbs were tested for FcγR binding at a range of 200 to 1.5625 ng/mL.

### Antibody-dependent NK cell activation

Antibody-dependent NK cell activation (ADNKA) assesses antigen-specific antibody-mediated NK cell activation against protein-coated plates. ELISA plates were coated with Stabilized SARS-CoV-2 spike trimer at a concentration of 300 ng/well, incubated for 2 hours at room temperature, and then blocked with 5% BSA-PBS overnight at 4˚C. Diluted antibody (diluted in PBS) was added to the antigen coated plates, and unbound antibodies were washed away. NK cells, purified from healthy blood donor leukopaks using commercially available negative selection kits (StemCell EasySep Human NK Cell Isolation Kit, Stem Cell Technologies, Cambridge, MA) were added to the plates at 5x10^4^ cells/well in R10 supplemented with anti-CD107a PE-Cy5 (BD Biosciences, 555802), GolgiPlug (BD Biosciences, 555029) and GolgiStop (BD Biosciences, 554724), and incubated for 5 hours at 37˚C. Following the incubation, NK cells were stained for the surface markers with anti-CD56 PE-Cy7, anti-CD16 APC-Cy7 and anti-CD3 Pacific Blue (BD Biosciences, 557747, 557758, 558124). NK cells were fixed and permeabilized with Fix&Perm cell permeabilization kit (Invitrogen). Cells were incubated with anti-MIP1β PE and anti-IFNγ FITC (BD Biosciences, 550078, 340449) to stain for intracellular markers. Fluorescence was acquired on a Stratedigm S1000EON. The data are reported as the percent of cells positive for each of the activation markers (CD107a, IFN-γ and MIP-1β). Each sample was tested with at least two different NK cell donors, with all samples tested with each donor. The mAbs were tested for ADNKA activity at a range of 20 μg/mL to 9.1449 ng/mL.

### Antibody-dependent cellular cytotoxicity

Antibody-dependent cellular cytotoxicity (ADCC) tests the ability of antigen-specific antibodies to recruit NK cell lytic activity. Target cells were biotinylated (EZ-Link Sulfo-NHS-LC-Biotin) and stained with one of two dyes (half with CellTrace Violet and half with CellTrace Far Red, ThermoFisher, Waltham, MA). The stained cells were then pulsed with either streptavidin-conjugated Stabilized SARS-CoV-2 spike trimer or left unpulsed. Diluted antibody (diluted in PBS) was added to a 1:1 mixture of the target cells stained with each dye. NK cells purified from healthy blood donor leukopaks using commercially available negative selection kits (StemCell EasySep Human NK Cell Isolation Kit) were added and incubated for 4 hours. Following this incubation, the cells were stained with a viability dye (BioLegend Zombie Green Fixable Viability Kit, BioLegend, San Diego, CA). Lysis was measured by flow cytometry, and lysis was reported as the percent of dead (viability dye positive) antigen-coated cells. The mAbs were tested for ADCC activity at a range of 1 μg/mL to 0.45725 ng/mL.

### Antibody-dependent complement deposition

Antibody-dependent complement deposition (ADCD) assesses the recruitment of complement component C3b on the surface of antigen-coupled beads. Stabilized SARS-CoV-2 spike trimer was covalently coupled to Carboxy-modified fluorospheres beads (Molecular Probes, F8815) via a two-step carbodiimide reaction. The beads were activated for 30 minutes at room temperature using 100 mM monobasic sodium phosphate, pH 6.2 with 5 mg/mL Sulfo-NHS (N-hydroxysulfosuccinimide, Pierce, A39269) and 5 mg/mL ethyl dimethylaminopropyl carbodiimide hydrochloride (EDC), then washed with 50 mM MES, pH 5.0, and incubated with 40 μg of antigen in 50 mM MES, pH 5.0 for two hours on a rotator. The coupled beads were blocked 5% BSA-PBS. After blocking, coupled beads were washed with 0.1% BSA-PBS, resuspended in 0.1% BSA-PBS, and stored at 4˚C. Diluted antibody (diluted in PBS) was added to coupled beads and incubated for 2 hours at 37 ˚C, and then washed with PBS. Commercially available guinea pig complement was then added as a source of complement (Sigma, G9774) and diluted 1:60 in GVB (Boston BioProducts, IBB-300X). Following a 50 minute incubation at 37˚C, the complement was washed away with 15mM EDTA in PBS, and a FITC-conjugated mAb specific for guinea pig C3 MP Biomedicals, 0855385) was added for 20 minutes. Complement deposition was measured using a Stratedigm S1000EON and is reported as the median fluorescent intensity of FITC. The mAbs were tested for ADCD activity at a range of 67 μg/mL to 30.636 ng/mL.

### Antibody-dependent cellular phagocytosis

Antibody-dependent cellular phagocytosis (ADCP) assesses the ability of antibodies to induce phagocytosis of antigen-functionalized fluorescent beads by monocytes via Fc receptors. Stabilized SARS-CoV-2 spike trimer was covalently coupled to Amine-modified fluorospheres beads (Molecular Probes, F8765) via a two-step carbodiimide reaction. The beads were activated for 30 minutes at room temperature using 100 mM monobasic sodium phosphate, pH 6.2 with 5 mg/mL Sulfo-NHS (N-hydroxysulfosuccinimide, Pierce, A39269) and 5 mg/mL ethyl dimethylaminopropyl carbodiimide hydrochloride (EDC), then washed with 50 mM MES, pH 5.0, and incubated with 40 μg of antigen in 50 mM MES, pH 5.0 for two hours on a rotator. The coupled beads were blocked 5% BSA-PBS. After blocking, coupled beads were washed with 0.1% BSA-PBS, resuspended in 0.1% BSA-PBS, and stored at 4˚C. Diluted antibody (diluted in PBS) was added to coupled beads and incubated for 4 hours at 37˚C, and then washed with PBS. THP-1 cells were added at a concentration of 7.5x10^4^ cells/mL and phagocytosis was allowed to proceed overnight at 37˚C. The cells were then fixed, and the extent of phagocytosis was measured using a Stratedigm S100EON. The data are reported as a phagocytic score, which takes into account the proportion of effector cells that phagocytosed and the degree of phagocytosis using the equation:

$phagocytic\;score=\frac{{percentage\;of\;FITC}^{+}\;cells\times {gMFI\;of\;FITC}^{+}\;cells}{1,000}$. The mAbs were tested for ADCP activity at a range of 5 μg/mL to 2.28624 ng/mL.

### Antibody-dependent neutrophil phagocytosis

Antibody-dependent neutrophil phagocytosis (ADNP) assesses the ability of antibodies to induce the phagocytosis of antigen-coated targets by primary neutrophils. Stabilized SARS-CoV-2 spike trimer was covalently coupled to Amine-modified fluorospheres beads (Molecular Probes, F8765) via a two-step carbodiimide reaction. The beads were activated for 30 minutes at room temperature using 100 mM monobasic sodium phosphate, pH 6.2 with 5 mg/mL Sulfo-NHS (N-hydroxysulfosuccinimide, Pierce, A39269) and 5 mg/mL ethyl dimethylaminopropyl carbodiimide hydrochloride (EDC), then washed with 50 mM MES, pH 5.0, and incubated with 40 μg of antigen in 50 mM MES, pH 5.0 for two hours on a rotator. The coupled beads were blocked 5% BSA-PBS. After blocking, coupled beads were washed with 0.1% BSA-PBS, resuspended in 0.1% BSA-PBS, and stored at 4˚C. Diluted antibody (diluted in PBS) was added to coupled beads and incubated for 2 hours at 37˚C, and then washed with PBS. Primary neutrophils isolated from healthy blood donors using negative selection (StemCell EasySep Direct Human Neutrophil Isolation Kit) at a concentration of 1.25x10^5^ cells/mL, and phagocytosis was allowed to proceed for 30 minutes at 37˚C. The cells were then stained with the neutrophil marker CD66b (Pacific Blue conjugated anti-CD66b; BioLegend, 305112), washed and fixed, and the extent of phagocytosis was measured by flow cytometry. The data are reported as a phagocytic score calculated using the equation above. Each sample was run in biological duplicate using neutrophils isolated from two distinct donors. The mAbs were tested for ADNP activity at a range of 300 μg/mL to 137.17 ng/mL.

### Antibody-dependent mucin binding

Antibody-dependent mucin binding (ADMB) measures the capacity of antibodies to trap pathogens in mucus proteins. Recombinant MUC5B or MUC5A/C (MyBioSource), the predominant secreted mucins in the lung, were used to coat ELISA plates at a concentration of 10 ng/well. Diluted antibody (diluted in PBS) was added and incubated for 2 hours at room temperature, and unbound antibodies were washed away with PBS-Tween. The bound antibodies were detected using an anti-human IgG antibody conjugated to HRP and TMB detection reagents. The data are reported as the background (no antibody)–subtracted OD. The mAbs were tested for mucin binding at a range of 2 mg/mL to 914 ng/mL.

### Antibody-dependent enhancement of infection

The antibody-dependent enhancement of infection (ADEI) assay measures the capacity of antibodies to facilitate the infection of FcγR-expressing cells. Antibodies were diluted in cell culture medium, and SARS-CoV-2 spike protein-pseudotyped lentiviruses encoding firefly luciferase were added. The antibodies and viruses were incubated for 1 hour, after which Raji cells (a B cell line that naturally expresses FcγR2) were added. The cultures were incubated for 24 hours, after which the culture medium was removed, and luciferase assay reagent was added. Following a two-minute incubation to allow for cell lysis, the supernatant was transferred to a black 96 well plate, and luciferase activity was measured using a luminometer. The data are reported as the relative infection rate compared to infection in the absence of antibody. The mAbs were tested for ADEI activity at a range of 6 μg/mL to 11.72 ng/mL.

## Results

### Monoclonal antibody binding to spike protein

Previous studies have demonstrated that the anti- SARS-CoV-2 antibodies bound to the spike protein also inhibited the binding to the ACE-2 receptor [50,51]. As shown in Tables 2 and S1, the addition of the YTE mutations or the YTE mutations in combination with the LALA mutations did not have any adverse effects on the antibodies ability to bind to the S1 domain of the spike protein or the RBD of the spike. As expected, none of the antibodies bound to the S2 domain of the spike protein. The combination of MAB2130 and MAB2381 did not appear to show any synergism to binding to S1 or RBD.

**Table 2:** ELISA binding results to different spike protein variants.

Antibody Description	S1 Binding EC50 ± Std Deviation (ng/mL)	RBD Binding EC50 ± Std Deviation (ng/mL)	S2 Binding EC50 (ng/mL)
mAb2130 WT	1.98 ± 0.04	2.68 ± 0.94	ND
mAb2130 YTE	15.53 ± 4.731	6.76 ± 5.33	ND
mAb2130 YTE-LALA	14.82 ±5.21	11.33 ± 2.01	ND
mAb2381 WT	1.50 ± 0.16	7.41 ± 0.37	ND
mAb2381 YTE	9.60 ± 2.39	8.53 ± 0.43	ND
mAb2381 YTE-LALA	10.73 ± 2.73	7.38 ± 1.26	ND
mAb2130 + mAb2381 YTE/LALA (ADM03820)	14.63 ± 2.95	7.80 ± 0.39	ND
mAb2130 + mAb2381 YTE (ADM03826)	12.69 ± 3.98	10.40 ± 0.52	ND

WT: Wild type sequence.

ND: No binding detected.

### Microneutralization potency studies with monoclonal antibodies

Previous studies have shown that the anti-SARS-CoV-2 antibodies did neutralize and weaken the ability of the virus to infect cells [50,51]. Results from the microneutralization assays are shown in Tables 3 and S2.

**Table 3:** Microneutralization analysis on various antibodies.

Antibody Sample	IC50 (ng/mL)	%CV
MAB2130 WT	9	NA^1^
MAB2130 YTE	17	34%
MAB2130 YTE-LALA	11	48%
MAB2381 WT	43	NA^1^
MAB2381 YTE	20	37%
MAB2381 YTE-LALA	15	41%
WT DP	<7.5	NA
YTE DP	11	7%
YTE-LALA DP	6	66%

YTE DP: MAB2130 YTE combined with MAB2381 YTE (ADM03826).

YTE-LALA DP: MAB2130 YTE-LALA combined with MAB2381 YTE-LALA (ADM03820).

Multiple lots were generated for each antibody and combination. Values given are mean values with the correlation of variation.

^1^ Not Applicable. These samples were tested once and were similar to previously published results [50,51]. YTE and YTE LALA variants were tested using a validated assay.

### Virus neutralization studies

**Fig 1:**
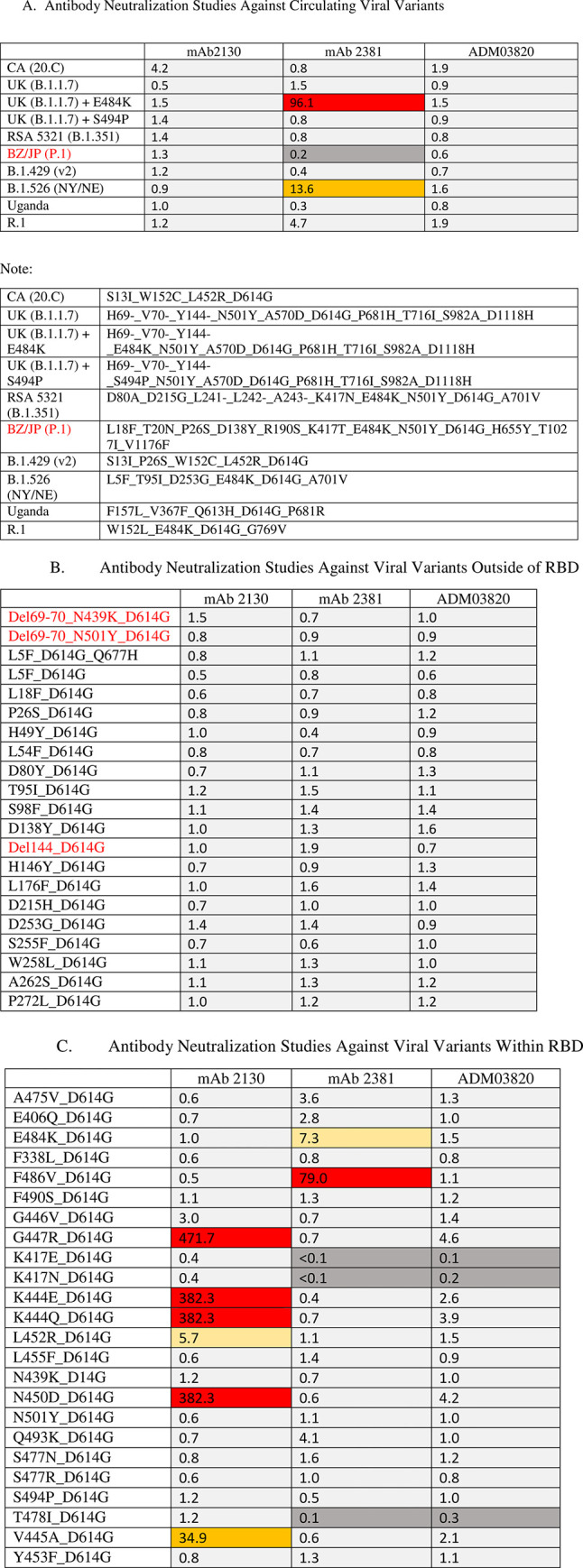
Viral neutralization studies. The results of the viral variant testing. Heat map represents activity as measured by the average ratio of the mutation IC50/wild type (D614G). Ratios are provided in the Figures. The average is calculated from multiple experiments so specific mutation IC50 and WT IC50 are not provided. IC50 (dark grey represents <0.3; light grey represents 0.3–5.0; yellow represents 5.0–10.0; orange represents 10.0–50.0; red represents >50.0). (A) Neutralization results against VOCs; (B) Neutralization results against a panel of mutant variants of the non-RBD, (C) Neutralization results against a panel of mutant variants of the RBD.

The pseudovirus assay was used to assess the efficacy of MAB2130 YTE-LALA, MAB2381 YTE-LALA or ADM03820 against various SARS-CoV-2 variants. As shown in Fig 1A, none had an adverse effect on the neutralization ability of MAB2130 YTE-LALA and only one variant (UK (B.1.1.7) + E484K) had a major adverse effect on the efficacy of MAB2381 YTE-LALA. The B.1.526 (NY/NE) variant had a modest effect on the efficacy of MAB2381 YTE-LALA. None of the viral variants with mutations in the non-RBD regions had any adverse effects on the neutralization of the antibodies in combination (Fig 1B). Four of the viral variants that were evaluated that represented changes in the RBD region abrogated the neutralization effects of MAB2130 YTE-LALA and two variants modestly abrogated the efficacy of MAB2130 YTE-LALA (Fig 1C). In a similar fashion, only one variant blocked the neutralization of MAB2381 YTE-LALA (F486V_D614G) and a single variant (E484K_D614G) modestly inhibited the neutralization effects of MAB2381 YTE-LALA (Fig 1C). The E484K_D614G variation is also found in the UK (B.1.1.7) + E484K variant. The other variant that inhibited the neutralization of MAB2381 YTE-LALA is not normally found in any of the circulating viral variants. It is interesting to note that none of the variants were able to have any effect on both antibodies. (Fig 1A–1C).

### Functional activity of monoclonal antibodies and Fc variants

**Fig 2:**
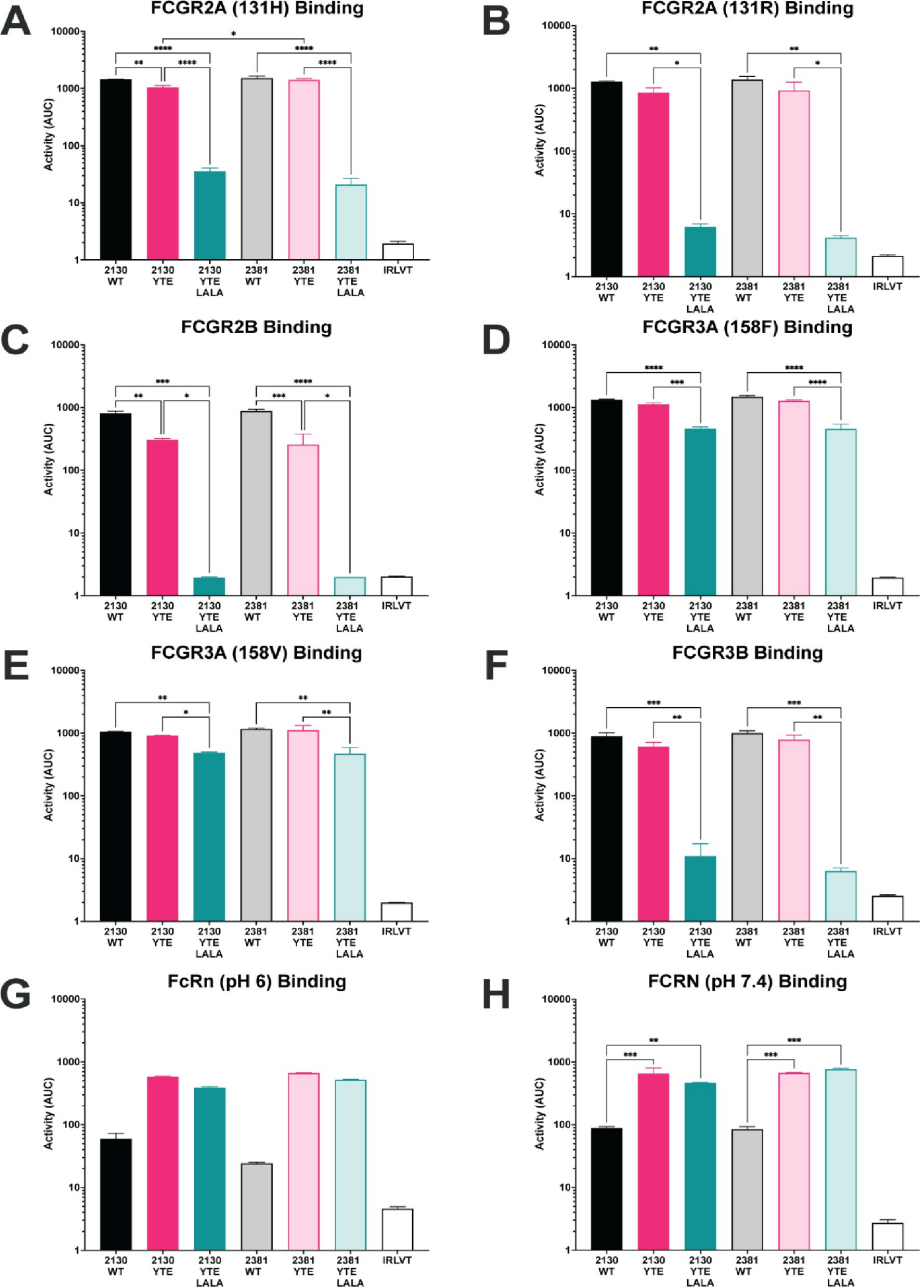
Binding of the monoclonal antibody variants to Fc receptors. The bar graphs present the overall binding of the monoclonal antibody variants to the indicated Fc receptor represented as the area under the curve calculated from the dilution curves presented in S1 Fig. Data are presented as the mean AUC ± standard deviation from two independent experiments. (A) FCGR2A 131H; (B) FCGR2A 131R; (C) FCGR2B; (D) FCGR3A 158F; (E) FCGR3A 158V; (F) FCGR3B; (G) FCRN pH6; (H) FCRN pH 7.4. The Ebola virus GP–specific antibody KZ52 was used as the irrelevant antibody. Significance was calculated using a one-way ANOVA with post hoc Holm-Šídák’s multiple comparisons test. *: p ≤ 0.05; ** p < 0.01; *** p < 0.001; **** p < 0.0001.

Extra-neutralizing antibody functions are mediated via interactions between the Fc domain of the antibody and Fc receptors found on the surface of all innate immune cells, with FcγR2A predominantly expressed on the surface of phagocytic cells (e.g., monocytes, macrophages, neutrophils); FcγR2B highly expressed on B cells and basophils, with weaker expression detected on monocytes and follicular dendritic cells; FcγR3A highly expressed on NK cells with weaker expression on macrophages and T cells; and FcγR3B found predominantly on myeloid cells [53]. As a first step to evaluate the functional activity of the various monoclonal antibodies, binding to the Fc receptors was evaluated using a Luminex-based assay (Figs 2 and S1) [54]. When on a wild type backbone, MAB2130 and MAB2381 demonstrated similar binding to all of the tested Fc receptors including both FcγR2A variants, (Figs 2A, 2B, S1A and S1B), FcγR2B (Figs 2C and S1C), both FcγR3A variants, (Figs 2D, 2E, S1D and S1E) and FcγR3B (Figs 2F and S1F). When expressed on the YTE backbones, binding of each antibody to FcγR2A, FcγR2B, FcγR3A, and FcγR3B was reduced when compared to the wild type IgG1 backbone; however, this reduction was largely not statistically significant (Figs 2A–2F and S1A–S1F). By contrast, binding of the antibodies to the Fc receptors was nearly completely ablated when expressed on the YTE-LALA variants, with only residual binding to FCGR3A observed (Figs 2A–1F and S1A–S1F). FCRN is expressed by endothelial cells and circulating monocytes and is important for the recycling of serum IgG, extending its serum half-life. As FCRN binding to IgG is pH dependent (FCRN binds to IgG tightly at acidic pH and weakly at physiologic pH), FCRN binding was tested at both pH 6 and pH 7.4 (Figs 2G, 2H, S1G and S1H)). Across the dilutions tested, there was no difference in the binding of any of the antibodies on an IgG1 backbone to FCRN at both pHs tested. While there was no difference in the binding of the two mAbs on the YTE backbone to FCRN at either pH, as expected, binding to FCRN was increased for each mAb on the YTE and YTE LALA backbones relative to its wild type IgG1 counterpart.

**Fig 3:**
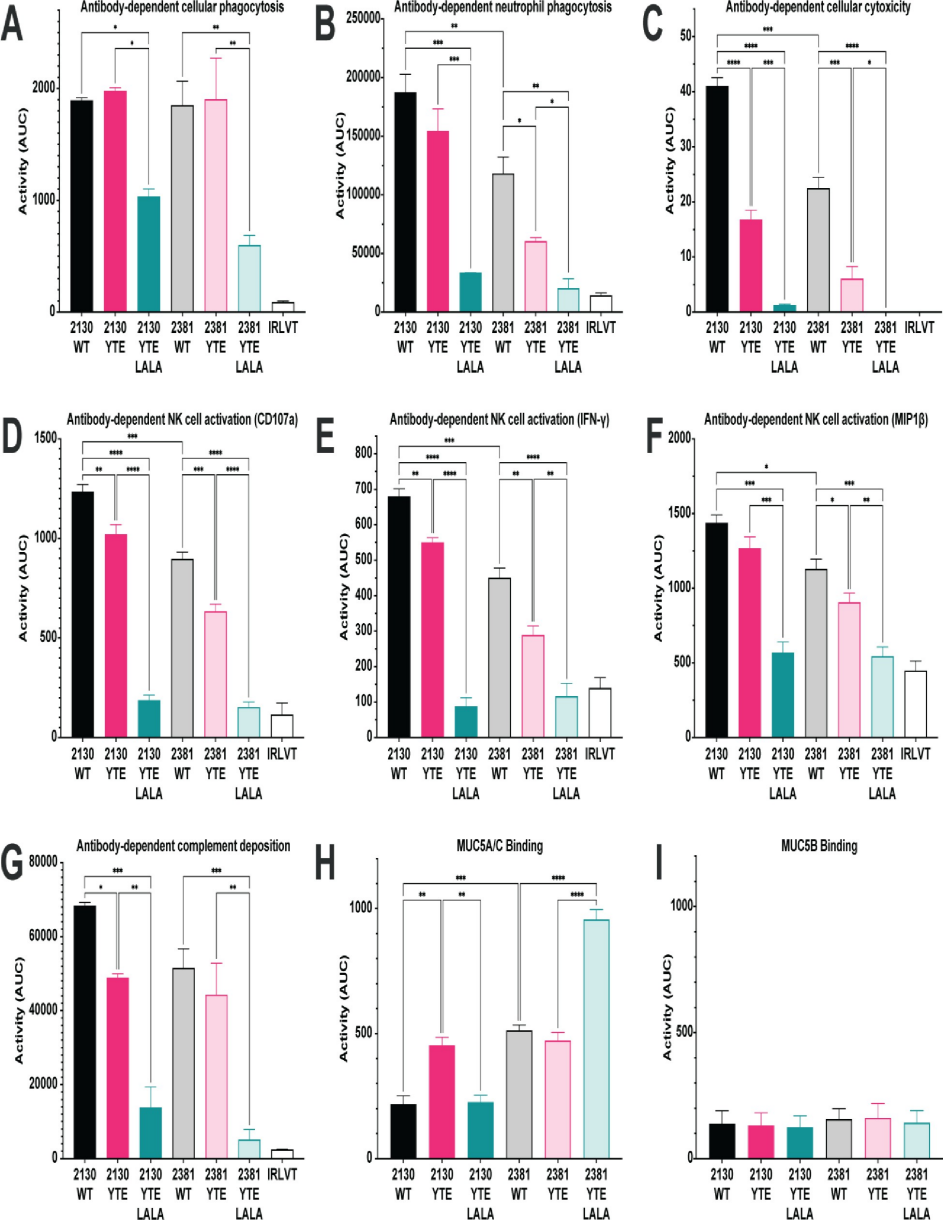
Extra-neutralizing functional activity of the monoclonal antibody variants. The data are presented both as bar graphs representing the overall activity of the monoclonal antibody variants in the indicated functional assays represented as the area under the curve calculated from the individual dilution curves presented in S2 Fig. Data are presented as the mean AUC ± standard deviation from two independent experiments. For assays using primary cells, cells isolated from two independent donors were used. (A) Antibody-dependent cellular phagocytosis; (B) antibody-dependent neutrophil phagocytosis; (C) antibody-dependent cellular cytotoxicity; (D–F) antibody-dependent NK cell activation; (G) antibody-dependent complement deposition; (H) antibody-dependent mucin (MUC5A/C) binding; (I) antibody-dependent mucin (MUC5B) binding. The Ebola virus GP–specific antibody KZ52 was used as the irrelevant antibody. Significance was calculated using a one-way ANOVA with post hoc Holm-Šídák’s multiple comparisons test. *: p ≤ 0.05; ** p < 0.01; *** p < 0.001; **** p < 0.0001.

While only limited differences in binding to FcγRs between the two monoclonal antibodies were observed in the binding assays, largely centered around binding to FCGR3A, these differences could translate into differences in the functional activity of the various monoclonal antibodies (Figs 3 and S2) [55,56].

Both MAB2130 and MAB2381 demonstrated strong ADCP activity when on a wild type IgG1 or the YTE backbone (Figs 3A and S2A). While not completely eliminating ADCP activity, the YTE-LALA Fc variant of both antibodies demonstrated significantly reduced ADCP activity, reducing the ADCP activity to approximately half of the activity observed on a wild type or YTE backbone. Both MAB2130 and MAB2381 demonstrated strong ADNP activity when on a wild type IgG1 backbone (Figs 3B and S2B), with MAB2130 demonstrating significantly increased ADNP activity relative to MAB2381. However, both antibodies demonstrated reduced ADNP activity when on the YTE backbone, and the YTE-LALA variant significantly reduced the ADNP activity of both antibodies reducing it to approximately 15% of the ADNP activity observed on a wild type IgG1 backbone.

In addition to driving phagocytosis of antibody-opsonized targets, antibodies can also harness the lytic potential of NK cells to drive the clearance of infected cells and pathogens. Both MAB2130 and MAB2381 demonstrated strong ADCC activity when expressed on the wildtype IgG1 backbone (Figs 3C and S2C), with MAB2130 again demonstrating significantly increased ADCC activity relative to MAB2381. Similar to ADNP activity, the YTE mutations significantly reduced the ADCC activity of both mAbs, reducing the ADCC activity to half of what is seen on a wild type IgG1 backbone. ADCC activity was the functional activity that was most dramatically impacted by the YTE-LALA mutations, with ADCC activity almost completely ablated on the YTE-LALA backbone. ADCC activity was reduced to approximately 5% of the activity observed on a wild-type backbone and was similar to the ADCC activity of an irrelevant mAb. Beyond direct killing of antibody-opsonized target cells, following activation, NK cells can release many different chemokines (e.g., MIP-1β) as well as pro-inflammatory cytokines (e.g., IFN-γ and TNF-α). These chemokines and cytokines function to recruit additional cells to sites of infection/inflammation and to enhance the immune response of the recruited cells. Across all three measures of ADNKA activity (NK cell degranulation [CD107a expression] cytokine secretion [IFN-γ], and chemokine secretion [MIP-1β]), both MAB2130 and MAB2381 demonstrated high levels of ADNKA activity (Figs 3D–3F and S2D–S2F), with MAB2130 demonstrating significantly increased activity when compared with MAB2381. Similar to the effect on ADCC activity, the YTE mutation similarly reduced the ADNKA activity of both antibodies with an average decrease of 25% observed across the panel of antibodies and detectors. Similar to ADCC activity, ADNKA activity was nearly completely eliminated on the YTE-LALA backbone.

In addition to activating FcγR-expressing innate immune cells, antibodies can also activate the classical complement cascade to eliminate infected cells and pathogens. While MAB2130 demonstrated increased ADCD activity relative to MAB2381, particularly at limiting antibody concentrations (Figs 3G and S2G), there was no significant difference in the overall ADCD activity between the two antibodies. While ADCD activity was universally reduced on the YTE backbone, the two antibodies demonstrated differential sensitivity to the mutations. While MAB2130 was highly sensitive to the effects of this mutation on ADCD activity, displaying approximately 65% of the activity of the wild type IgG1 counterpart, MAB2381 was relatively insensitive to the effects, demonstrating only a 20% reduction in ADCD activity relative to the wild type variant. ADCD activity was also significantly reduced on the YTE-LALA backbone. MAB2381 demonstrated ADCD activity similar to an irrelevant mAb, whereas the ADCD activity of MAB2130 was reduced to 20% of that observed on the wild type IgG1 backbone.

Finally, all mucosal surfaces are lined with a thick layer of mucus that provides a protective physical barrier by trapping pathogens. Anti-microbial peptides, immune proteins, and antibodies are present within mucus and can be bound to a lattice of heavily glycosylated mucin proteins that line the membranes [57]. Although the majority of the antibodies present in the mucosal surfaces are of the IgA isotype, considerable amounts of IgG isotypes may also be found and involved in immune surveillance. These antibodies have been shown to decrease the movement of viruses through the mucus, preventing infection of the underlying epithelium [58]. Focusing on the predominant mucins produced by the surface epithelium and by airway epithelial cells in culture [59], binding to MUC5A/C (Figs 3H and S2H) and MUC5B (Figs 3I and S2I) and was evaluated using an ELISA-based assay. While neither antibody demonstrated binding to MUC5B, both antibodies bound MUC5A/C although MAB2381 demonstrated significantly higher binding. Interestingly, unlike most of the functional activities, which were negatively impacted by the YTE mutations, the binding of MAB2130-YTE to MUC5A/C and MAB2381 YTE-LALA was increased compared to the wildtype IgG1 variant.

### Antibody-dependent enhancement of infection

Antibody-dependent enhancement of infection has been repeatedly demonstrated *in vitro* for human coronaviruses, including SARS and MERS [60,61]. Unlike traditional infection of cells, this infection occurs in the absence of the known cellular receptor for virus entry and is dependent on the expression of FcγRs on the surface of the target cell, traditionally FCGR-2A 2A for SARS CoV1 and MERS [23], and the presence of antibodies targeting the virus. Importantly, recent evidence suggests that some SARS CoV2–specific antibodies, including both monoclonal antibodies [62,63] and polyclonal serum from convalescent subjects [63], can also mediate this effect, albeit in an FcγR 2B-dependent manner. To determine if this panel of monoclonal antibodies could induce a similar effect, the full antibody panel was tested for their ability to drive the infection of ACE2-negative but FCGR-2A-positive Raji cells. Infection of these cells was not observed at any concentration of any of the mAbs (S3 Fig), suggesting that while some SARS CoV2–specific antibodies can mediate this type of infection, it is highly antibody specific and not all antibodies drive this effect.

## Discussion

Individuals who recover from certain viral infections typically develop virus-specific antibody responses that provide robust protective immunity against re-exposure. Since the emergence of SARS-CoV-2, several groups [64] have reported the isolation of neutralizing antibodies from survivors that target the spike protein and neutralize the virus [64–71]. A common observation across these studies is that the SARS-CoV-2 RBD is the target of potent neutralizing antibodies. In the current study, we evaluate the effects of a serum half-life extending mutation, YTE on the ability of four previously identified anti-SARS-CoV-2 antibodies to bind to the spike protein, neutralize viral infections in cell culture as well as on the effector function of each antibody. A similar profile of studies was performed with a combination of the YTE mutation with an effector function blocking mutation, LALA. We also evaluated the ability of a combination of two mAb to bind to the spike protein and neutralize the virus.

Overall, the individual mAbs have similar functional activities with respect to binding to the S1 and RBD domains of the spike protein, neutralizing viral infections in a cell culture model and to inducing all the Fc effector functions. The binding affinities and microneutralization results of MAB2130 and MAB2381 are comparable to the REGN-COV2 antibodies [72,73] and LY-Cov-555 plus Ly-CoV-016 [74]. In general, MAB2130 was the most potent in its ability to bind to the S1 and RBD domains of the spike protein as well as in neutralizing viral infections. Of particular note, a combination of two antibodies (MAB2130 and MAB2381) did not result in a decreased ability of the antibodies to bind to the spike protein domains or neutralize viral infection of the cells. This is probably the result that these antibodies do not appear to bind to the same epitopes on the spike protein [50,51]. It has been shown that MAB2130 appears to be able to bind to both the open and closed positions of the spike protein, whereas MAB2381 recognizes only the closed position of the spike protein [51]. In addition, MAB2130 has been shown to be protective in a SARS-CoV-2 infection model in BALB/c mice [50].

The antibodies alone were very effective in neutralizing most of viral variants that were evaluated. Only one of the circulation viral variants of concern was able to avoid neutralization by the MAB2381 YTE-LALA antibody alone (UKB.1.1.7) + E484K). There is another circulating virus (RSA 5321 B.1.351) that contains the same mutations at positions 501 and 484 as well as an additional mutation K417 that did not avoid neutralization by MAB2381 YTE-LALA. It is speculated that the additional mutation conferred a conformational change on the spike protein that allowed it to be recognized by MAB2381 YTE-LALA. One of the mutations that was found in that viral variant was also identified in our pseudovirus screening assay. However, in the pseudovirus screen, this mutation was only able to partially diminish the neutralizing effects of this antibody. Another mutation (F486V_D614G) was able to inhibit the neutralization of this antibody however, this mutation has not been found in circulating virus. Several viral mutations were also shown to diminish the neutralizing effects of MAB2130 YTE-LALA. None of the viral variants were able to block the neutralizing effects of both antibodies. This should not be surprising, as it has been shown that these antibodies bind to non-overlapping regions of the RBD. The most important aspect of this screening dataset is that none of the circulating viral variants or the viral mutations that were screened in this study were able to inhibit the neutralizing effects of the combination of the two antibodies, ADM03820).

Generally, MAB2130 was more functional than MAB2381 on the wild type IgG1 format in the Fc effector function screening demonstrating significantly increased ADNP, ADCC, and ADNKA activity relative to MAB2381. For antibodies that contain similar Fc regions and recognize the same protein target,albeit different epitopes on the protein [50,75], differences in antibody function are likely due to differences in fine antibody specificity (the precise epitope recognized by the antibody) or the angle at which the antibodies are binding to the antigen as changes in the geometry of the antibody:antigen interaction have the potential to change the accessibility of the Fc domain for interactions with FcRs.

In general, the mutations did not have any effects on an antibody’s ability to bind to the spike protein or neutralize the virus. Our results also demonstrate that the presence of the YTE-LALA mutations did not inhibit ability of MAB 2130 or MAB2381 to bind to the spike protein or neutralize the virus in a similar fashion as observed previously [43]. This was not unexpected as these mutations have not been shown to alter the epitope binding of the parent antibody. The YTE mutations (M252Y/S254T/T256E) were designed to decrease the rate of Fc and FcRn mediated serum clearance and enhance the overall serum half-life of the antibody [47]. Inclusion of these three mutations resulted in a four-fold increase in serum half-life as compared to a wild type antibody [48]. The pharmacokinetic profile of an IgG1 possessing the YTE mutations was determined in a Phase 1 dose escalation study and was shown to increase the serum half-life to 80–112 days [49]. In a second study, the YTE mutation was introduced into motavizumab and was shown to have functional therapeutic antibody present in the serum 240 days after antibody infusion [76].

The YTE Fc domain is a serum half-life–extending IgG1 variant [76] that was selected for its optimized binding to FCRN. As expected, all tested mAbs demonstrated increased binding to FCRN compared to their IgG1 counterparts. Interestingly, this increased interaction was observed at both acidic (pH 6) and neutral (pH 7.4) conditions. As increased antibody half-life is tied to FCRN-dependent recycling of IgG, antibodies with an increased half-life should have increased binding to FCRN at acidic pH and similar binding at neutral pH. In previous studies of the impact of the YTE mutation on FCRN binding [77], reduced binding to FCRN at physiologic pH was only observed at low antibody and FCRN densities. However, the assay used here was carried out using a high density of antigen on the surface of beads and high-density tetramerized FCRN as a detector reagent. Therefore, it is likely that the increased biding of the YTE variants at physiologic pH is likely due to the methodology used in the FCRN binding assay.

While the YTE variant was selected for its increased interaction with FCRN, alterations in the Fc domain of an antibody can result in off-target effects where binding to other Fc receptors is enhanced or compromised, potentially altering the Fc functionality of the antibody, and antibodies on the YTE backbone have demonstrated lower ADCC activity [78] than antibodies on a wild type IgG1 Fc domain. For both MAB2130 and MAB2381, binding to FCGR2A and FCGR2B was significantly decreased on the YTE backbone when compared to the wild type IgG1. This reduced binding had functional consequences as the YTE variants demonstrated reduced ADNP, ADCD, ADCC and ADNKA activity compared to the wild type IgG1 versions. Combined, these results suggest that beyond impacting binding to FCRN, the YTE mutation can impact binding to other FcγRs, which has the potential to impact the functionality of the antibody; however, these effects may be dependent on the Fab of the antibody.

Recent work has begun to determine the impact of stability- and functionality-enhancing/reducing mutations on the structure and conformation of the Fc domain. FcγRs interact with the CH2 domain of the antibody Fc, and small changes in the conformation of the CH2 domain can dramatically impact FcγR binding. The YTE variants have been previously shown to alter the thermal stability [79] of an antibody and, importantly, recent work has further demonstrated that the YTE mutations alters the conformation and flexibility of the Fc domain, particularly around the CH2 domain of the Fc [80] suggesting that the altered functionality of the YTE variants is not surprising. While altered binding to FcγR can be Fab dependent, reduced binding of both MAB2130 and MAB2381 on the YTE backbone was observed in the present study. Beyond direct binding of the Fab domains to FcRs [81], the Fab domain can direct the binding of monoclonal antibodies to their target protein in such a way that the conformational flexibility of the Fc domain is restricted and that the conformational changes induced by the YTE mutation further restrict the conformational space that the Fc domain resides in, ultimately limiting its accessibility to the incoming FcγR and impacting antibody function. This was not observed in the antibodies that were investigated in this report.

We are focused on reducing the antibody Fc function to diminish the possibility of the Fc region binding to cells that do not express the ACE2 receptor and to modulate the function of the effector cells that normally bind the Fc receptor. The L234A/L235A mutations ablate antibody functionality by reducing the interaction between the Fc domain of the antibody and FcγRs and are among the most common point mutations used to disrupt Fc receptor binding. These mutations have been shown to eliminate detectable binding to several Fc receptors [41]. The LALA mutations have also been shown to reduce ADCC activity mediated by PBMCs and nearly ablated ADCC mediated by monocytes [82]. Our data supports these previous observations. The LALA mutations have been tested in Phase 1 clinical trials with minimal adverse reactions and was able to reverse allograft rejection in 6 of 7 individuals with renal allograft implants [42].

As expected, antibodies on the YTE LALA backbone were largely non-functional in the Fc effector function assays, with only minimal ADCP activity detected. Although antibodies on the YTE-LALA backbone were largely non-functional, MAB2819 YTE-LALA demonstrated increased binding to MUC 5A/C relative to MAB2819 on a wild type backbone (but reduced compared to MAB2819 YTE), suggesting that while this Fc variant may ablate interaction with FcγRs, it does not necessarily impact the binding of the Fc domain to other Fc-binding proteins. The antibody:mucin interaction is largely mediated by the Fc domain of the antibody. As changes in antibody glycosylation have been shown to impact binding of antibodies to mucins, it is not surprising that point mutations that may impact the overall structure of the Fc domain may similarly impact binding to mucins.

Antibody-dependent infection of cells lacking the traditional cell surface receptor for coronaviruses (ACE2 for SARS-CoV-1 and SARS-CoV-2, and DPPR from MERS-CoV) has been reported previously [3,8,9,23]. Recently, antibody-dependent infection of ACE2-negative cells by SARS-CoV-2-pseudotyped reporter virus has been reported (https://www.biorxiv.org/content/10.1101/2020.07.26.222257v1) suggesting that this may be a general phenomenon for all coronaviruses. However, only one of the mAbs tested in these previous studies demonstrated this activity, suggesting that not all mAbs may mediate this effect. Using an assay similar to that used to demonstrate this effect for SARS-CoV-2, none of the antibodies tested here demonstrated any antibody-dependent infection of ACE2-negative, FcγR2B-positive Raji cells. These results suggest that while some mAbs can potentially drive antibody-dependent infection of ACE2-negative cells, potentially limiting their therapeutic use, the antibodies in this study did not induce this activity.

The combination product, ADM03820 is currently being evaluated in Phase 1 clinical studies in healthy human volunteers. The results presented in this study support the potential utility of the combination of the YTE and LALA variants to the antibody cocktail that is currently being investigated as an anti-SARS-CoV-2 antibody drug product for COVID-19 patients.

In summary, the present studies demonstrated the utility of selected modifications (mutations) to enhance the utility of therapeutic mAb, in particular those directed against SARS-CoV-2, although certainly any mAb intended for therapeutic applications for infectious disease should benefit from either, or both, of these modifications.

## Supporting information

S1 Fig Binding of the monoclonal antibody variants to Fc receptors. The binding of the antibody variants to the indicated Fc receptor is presented as a line graph demonstrating the binding across the range of antibody dilutions tested. The data are presented as the mean ± standard error from two independent experiments. (A) FCGR2A 131H; (B) FCGR2A 131R; (C) FCGR2B; (D) FCGR3A 158F; (E) FCGR3A 158V; (F) FCGR3B; (G) FCRN pH6; (H) FCRN pH 7.4. The Ebola virus GP–specific antibody KZ52 was used as the irrelevant antibody.
(DOCX)

S2 Fig Extra-neutralizing functional activity of the monoclonal antibody variants. The functional activity of the antibody variants is presented as a line graph demonstrating the functional activity across the range of antibody dilutions tested. The data are presented as the mean ± standard error. For assays using primary cells, cells isolated from two independent donors were used. (A) ADCP; (B) ADNP; (C) ADCC; (D) ADNKA CD107a (E) ADNKA IFN-gamma; (F) ADNKA MIP-1β; (G) ADCD; (H) ADMB MUC5AC; (I) ADMB MUC5B.
(DOCX)

S3 Fig Ability of the antibodies to drive the antibody-dependent, ACE2-independent infection of Raji cells. The ability of the monoclonal antibodies to drive the ACE2-independent infection of Raji cells is presented as the fold of background infection in the presence of the irrelevant monoclonal antibody. The data are presented as the mean ± standard error from two independent experiments. The positive control in this assay is serum collected from a convalescent individual; the Ebola virus GP–specific mAb KZ52 was used as an irrelevant control.
(DOCX)

S1 Table Supplemental ELISA binding results to different spike protein variants. (DOCX)

S2 Table Supplemental microneutralization analysis on various antibodies. (DOCX)
