# Supplementary figures and images for: Evaluation of strategies to modify Anti-SARS-CoV-2 monoclonal antibodies for optimal functionality as therapeutics
Source: PLoS One. 2022 Jun 3;17(6):e0267796. doi: 10.1371/journal.pone.0267796 (PMC9165815; doi:10.1371/journal.pone.0267796)

**S1 Figure: Binding of the Monoclonal Antibody Variants to Fc Receptors**


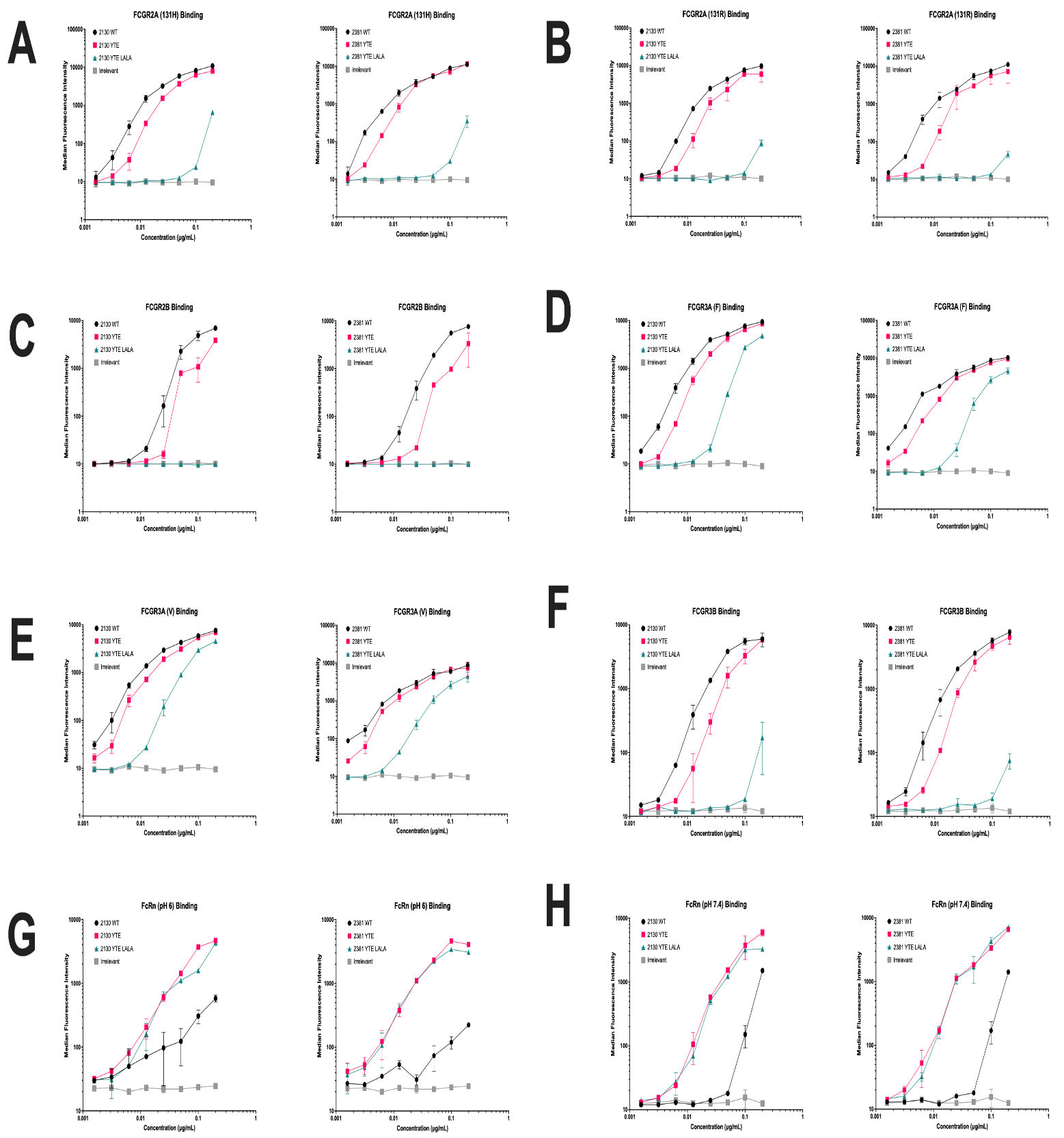

Supplement: S1 Fig — The binding of the antibody variants to the indicated Fc receptor is presented as a line graph demonstrating the binding across the range of antibody dilutions tested. The data are presented as the mean ± standard error from two independent experiments. (A) FCGR2A 131H; (B) FCGR2A 131R; (C) FCGR2B; (D) FCGR3A 158F; (E) FCGR3A 158V; (F) FCGR3B; (G) FCRN pH6; (H) FCRN pH 7.4. The Ebola virus GP–specific antibody KZ52 was used as the irrelevant antibody. (DOCX) [file pone.0267796.s001.docx]

**S2 Figure: Extra-Neutralizing Functional Activity of the Monoclonal Antibody Variants**


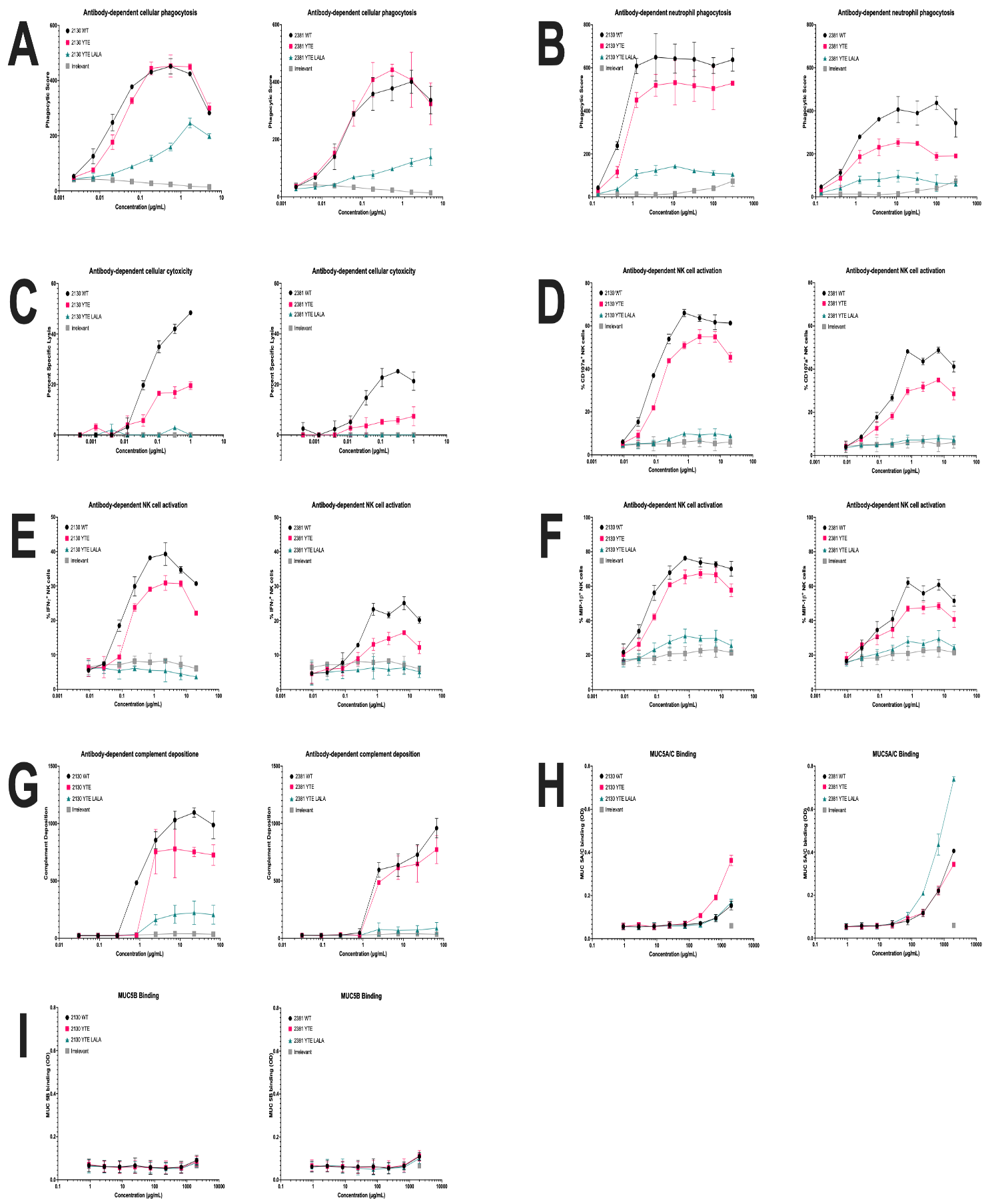

Supplement: S2 Fig — The functional activity of the antibody variants is presented as a line graph demonstrating the functional activity across the range of antibody dilutions tested. The data are presented as the mean ± standard error. For assays using primary cells, cells isolated from two independent donors were used. (A) ADCP; (B) ADNP; (C) ADCC; (D) ADNKA CD107a (E) ADNKA IFN-gamma; (F) ADNKA MIP-1β; (G) ADCD; (H) ADMB MUC5AC; (I) ADMB MUC5B. (DOCX) [file pone.0267796.s002.docx]
