# Supplementary material for: Evaluation of strategies to modify Anti-SARS-CoV-2 monoclonal antibodies for optimal functionality as therapeutics
Source: PLoS One. 2022 Jun 3;17(6):e0267796. doi: 10.1371/journal.pone.0267796 (PMC9165815; doi:10.1371/journal.pone.0267796)
Supplement: S3 Fig — The ability of the monoclonal antibodies to drive the ACE2-independent infection of Raji cells is presented as the fold of background infection in the presence of the irrelevant monoclonal antibody. The data are presented as the mean ± standard error from two independent experiments. The positive control in this assay is serum collected from a convalescent individual; the Ebola virus GP–specific mAb KZ52 was used as an irrelevant control. (DOCX) [file pone.0267796.s003.docx]

**S3 Figure:** **Ability of the antibodies to drive the antibody-dependent, ACE2-independent infection of Raji cells.**
