# Supplementary material for: Evaluation of strategies to modify Anti-SARS-CoV-2 monoclonal antibodies for optimal functionality as therapeutics
Source: PLoS One. 2022 Jun 3;17(6):e0267796. doi: 10.1371/journal.pone.0267796 (PMC9165815; doi:10.1371/journal.pone.0267796)
Supplement: S1 Table — (DOCX) [file pone.0267796.s004.docx]

**S1 Table: Supplemental ELISA Binding Results to Different Spike Protein Variants***

| Antibody | S1 Binding | | RBD Binding | |
| --- | --- | --- | --- | --- |
| mAb2130 WT | Dilution | OD450-630 | Dilution | OD450-630 |
|  | 250 | 2.21 | 500 | 2.21 |
|  | 125 | 2.12 | 250 | 2.45 |
|  | 62.5 | 2.13 | 125 | 2.40 |
|  | 31.25 | 2.16 | 62.5 | 2.31 |
|  | 15.625 | 1.90 | 31.25 | 2.31 |
|  | 7.812 | 1.87 | 15.625 | 2.26 |
|  | 3.906 | 1.56 | 7.812 | 2.03 |
|  | 1.953 | 1.11 | 3.906 | 1.74 |
|  | 0.977 | 0.65 | 1.953 | 1.26 |
|  | 0.488 | 0.36 | 0.977 | 0.80 |
|  | 0.244 | 0.21 | 0.488 | 0.53 |
| EC50 ng/mL) |  | 1.96 |  | 2.17 |
| mAb2130 YTE | 500 | 2.133 | 500 | 2.692 |
|  | 214 | 2.202 | 214 | 2.538 |
|  | 91.8 | 1.919 | 91.8 | 2.135 |
|  | 39.4 | 1.615 | 39.4 | 2.078 |
|  | 16.9 | 1.163 | 16.9 | 1.463 |
|  | 7.23 | 0.704 | 7.23 | 0.908 |
|  | 3.10 | 0.382 | 3.10 | 0.442 |
|  | 1.33 | 0.200 | 1.33 | 0.201 |
|  | 0.57 | 0.078 | 0.57 | 0.086 |
|  | 0.24 | 0.035 | 0.24 | 0.042 |
|  | 0.11 | 0.015 | 0.11 | 0.018 |
| EC50 ng/mL) |  | 15.9 |  | 14.29 |
| mAb2130 YTE-LALA | 500 | 1.519 | 500 | 2.384 |
|  | 214 | 1.296 | 214 | 2.393 |
|  | 91.8 | 1.285 | 91.8 | 2.171 |
|  | 39.4 | 1.106 | 39.4 | 2.064 |
|  | 16.9 | 0.855 | 16.9 | 1.701 |
|  | 7.23 | 0.504 | 7.23 | 0.951 |
|  | 3.10 | 0.286 | 3.10 | 0.568 |
|  | 1.33 | 0.146 | 1.33 | 0.257 |
|  | 0.57 | 0.096 | 0.57 | 0.140 |
|  | 0.24 | 0.044 | 0.24 | 0.061 |
|  | 0.11 | 0.034 | 0.11 | 0.031 |
| EC50 ng/mL) |  | 13.85 |  | 9.32 |
| mAb2381 WT | 250 | 2.31 | 500 | 2.835 |
|  | 125 | 2.26 | 214 | 2.778 |
|  | 62.5 | 2.29 | 91.8 | 2.764 |
|  | 31.25 | 2.14 | 39.4 | 2.383 |
|  | 15.625 | 2.13 | 16.9 | 2.147 |
|  | 7.812 | 2.00 | 7.23 | 1.380 |
|  | 3.906 | 1.77 | 3.10 | 0.803 |
|  | 1.953 | 1.27 | 1.33 | 0.501 |
|  | 0.977 | 0.84 | 0.57 | 0.217 |
|  | 0.488 | 0.49 | 0.24 | 0.107 |
|  | 0.244 | 0.28 | 0.11 | 0.043 |
| EC50ng/mL |  | 1.66 |  | 7.41 |
| mAb2381 YTE | 500 | 2.053 | 500 | 2.536 |
|  | 214 | 1.892 | 214 | 2.463 |
|  | 91.8 | 1.861 | 91.8 | 2.256 |
|  | 39.4 | 1.590 | 39.4 | 2.108 |
|  | 16.9 | 1.221 | 16.9 | 1.809 |
|  | 7.23 | 0.853 | 7.23 | 1.115 |
|  | 3.10 | 0.474 | 3.10 | 0.595 |
|  | 1.33 | 0.244 | 1.33 | 0.289 |
|  | 0.57 | 0.121 | 0.57 | 0.134 |
|  | 0.24 | 0.049 | 0.24 | 0.074 |
|  | 0.11 | 0.031 | 0.11 | 0.028 |
| EC50 ng/mL) |  | 11.08 |  | 8.53 |
| mAb2381 YTE-LALA | 500 | 2.397 | 500 | 2.501 |
|  | 214 | 2.030 | 214 | 2.458 |
|  | 91.8 | 2.181 | 91.8 | 2.335 |
|  | 39.4 | 1.961 | 39.4 | 2.234 |
|  | 16.9 | 1.530 | 16.9 | 2.025 |
|  | 7.23 | 1.004 | 7.23 | 1.311 |
|  | 3.10 | 0.585 | 3.10 | 0.797 |
|  | 1.33 | 0.322 | 1.33 | 0.436 |
|  | 0.57 | 0.162 | 0.57 | 0.209 |
|  | 0.24 | 0.084 | 0.24 | 0.092 |
|  | 0.11 | 0.047 | 0.11 | 0.042 |
| EC50 ng/mL) |  | 9.11 |  | 6.12 |
| mAb2130 + mAb2381 YTE-LALA (ADM03820) | 500 | 1.946 | 500 | 2.438 |
|  | 214 | 1.912 | 214 | 2.413 |
|  | 91.8 | 1.985 | 91.8 | 2.362 |
|  | 39.4 | 1.502 | 39.4 | 2.040 |
|  | 16.9 | 1.034 | 16.9 | 1.776 |
|  | 7.23 | 0.677 | 7.23 | 1.238 |
|  | 3.10 | 0.383 | 3.10 | 0.602 |
|  | 1.33 | 0.195 | 1.33 | 0.409 |
|  | 0.57 | 0.102 | 0.57 | 0.172 |
|  | 0.24 | 0.045 | 0.24 | 0.086 |
|  | 0.11 | 0.028 | 0.11 | 0.042 |
| EC50 ng/mL) |  | 15.10 |  | 7.80 |
| mAb2130 + mAb2381 YTE(ADM03826) | 500 | 2.484 | 500 | 2.792 |
|  | 214 | 2.432 | 214 | 2.745 |
|  | 91.8 | 1.853 | 91.8 | 2.187 |
|  | 39.4 | 1.927 | 39.4 | 2.295 |
|  | 16.9 | 1.438 | 16.9 | 1.793 |
|  | 7.23 | 0.919 | 7.23 | 1.065 |
|  | 3.10 | 0.447 | 3.10 | 0.697 |
|  | 1.33 | 0.238 | 1.33 | 0.261 |
|  | 0.57 | 0.094 | 0.57 | 0.149 |
|  | 0.24 | 0.041 | 0.24 | 0.064 |
|  | 0.11 | 0.026 | 0.11 | 0.027 |
| EC50 ng/mL) |  | 13.51 |  | 10.40 |

*These are a representative sample of the ELISA testing. Samples were repeated up to 10 times for each assay condition.
