# Supplementary material for: Evaluation of strategies to modify Anti-SARS-CoV-2 monoclonal antibodies for optimal functionality as therapeutics
Source: PLoS One. 2022 Jun 3;17(6):e0267796. doi: 10.1371/journal.pone.0267796 (PMC9165815; doi:10.1371/journal.pone.0267796)
Supplement: S2 Table — (DOCX) [file pone.0267796.s005.docx]

**S2 Table: Supplemental Microneutralization Analysis on Various Antibodies**

| Sample ID | Test Day | Operator 1 | Operator 2 | Operator 3 |
| --- | --- | --- | --- | --- |
| mAb 2130 YTE/LALA Lot Release | 1 | 15 | 20 | 15 |
|  |  | 14 | 18 | 18 |
|  |  | 18 | 16 | 14 |
|  |  | 19 | 16 | 14 |
|  | 2 | 9 | Not Reportable | 14 |
|  |  | <7 | Not Reportable | 7 |
|  |  | <7 | Not Reportable | 13 |
|  |  | 10 | 9 | 16 |
|  | 3 | 10 | 9 | <7 |
|  |  | 9 | 9 | 9 |
|  |  | <7 | 18 | <7 |
|  |  | <7 | 9 | <7 |
| mAb 2381 YTE/LALA Lot Release | 1 | 25 | <7 | 26 |
|  |  | 15 | 21 | 16 |
|  |  | 19 | 10 | 17 |
|  |  | 22 | 21 | 15 |
|  | 2 | 19 | 15 | 33 |
|  |  | 12 | 12 | 14 |
|  |  | 17 | 13 | 10 |
|  |  | 9 | 15 | 8 |
|  | 3 | 14 | 8 | 13 |
|  |  | 6 | 9 | 18 |
|  |  | 11 | 9 | 16 |
|  |  | 8 | 9 | 9 |
| ADM03820 Lot Release | 1 | <7 | Not Reportable | <7 |
|  |  | <7 | Not Reportable | <7 |
|  |  | <7 | Not Reportable | <7 |
|  |  | <7 | <7 | <7 |
|  | 2 | 8 | 8 | <7 |
|  |  | <7 | 8 | <7 |
|  |  | 14 | 12 | <7 |
|  |  | 10 | Not Reportable | <7 |
|  | 3 | 10 | <7 | <7 |
|  |  | 10 | <7 | <7 |
|  |  | 9 | 12 | 7 |
|  |  | 10 | <7 | 21 |
| mAb2130 YTE^1^  Lot Release | 1 | 23 | 12 |  |
|  | 2 |  | 15 |  |
| mAb2381 YTE^1^  Lot Release | 1 | 29 | 15 |  |
|  | 2 |  | 17 |  |
| ADM03826^1^ Lot Release | 1 | 10 | 11 |  |

Wild type antibodies were only tested once as the data corroborated previously published data.

^1^ YTE variants were tested using a validated assay method
